# Revising human-systems engineering principles for embedded AI applications

**DOI:** 10.3389/fnrgo.2023.1102165

**Published:** 2023-01-26

**Authors:** M. L. Cummings

**Affiliations:** Mechanical Engineering, Electrical and Computer Engineering, Computer Science, George Mason University, Fairfax, VA, United States

**Keywords:** human, autonomy, artificial intelligence, system engineering, requirements, testing, workforce development

## Abstract

The recent shift from predominantly hardware-based systems in complex settings to systems that heavily leverage non-deterministic artificial intelligence (AI) reasoning means that typical systems engineering processes must also adapt, especially when humans are direct or indirect users. Systems with embedded AI rely on probabilistic reasoning, which can fail in unexpected ways, and any overestimation of AI capabilities can result in systems with latent functionality gaps. This is especially true when humans oversee such systems, and such oversight has the potential to be deadly, but there is little-to-no consensus on how such system should be tested to ensure they can gracefully fail. To this end, this work outlines a roadmap for emerging research areas for complex human-centric systems with embedded AI. Fourteen new functional and tasks requirement considerations are proposed that highlight the interconnectedness between uncertainty and AI, as well as the role humans might need to play in the supervision and secure operation of such systems. In addition, 11 new and modified non-functional requirements, i.e., “ilities,” are provided and two new “ilities,” auditability and passive vulnerability, are also introduced. Ten problem areas with AI test, evaluation, verification and validation are noted, along with the need to determine reasonable risk estimates and acceptable thresholds for system performance. Lastly, multidisciplinary teams are needed for the design of effective and safe systems with embedded AI, and a new AI maintenance workforce should be developed for quality assurance of both underlying data and models.

## 1. Introduction

Complex systems, often with safety-critical implications like those in military and transportation systems, increasingly leverage artificial intelligence (AI) to enhance system performance. When faced with insufficient data, incomplete information and uncertain conditions, AI often cannot provide the necessary decision and/or action support and cannot rely on theory-based predictions to fill any reasoning gaps (Bishop, 2021), which could have catastrophic outcomes like the death of a pedestrian in the Uber self-driving accident in AZ in 2018 (Laris, 2018).

In comparison to other articles about machine learning, there has been relatively little discussion of how the inclusion of embedded AI could or should change how systems should be conceived, designed and testing, aka, the system engineering process. It is generally agreed that systems engineering approaches should consider how people and organizations, conceive, develop and manage complex systems, also known as human systems engineering (Weck et al., 2011). However, because of the increasing use of embedded AI across many facets of society, human systems engineering practitioners need to adapt to this new technology, which will require new touchpoints in the design and deployment of such systems.

With the increasing use of software across complex systems, agile software development practices have become the defacto standard (Crowder and Friess, 2013). While in theory the agile process involves users earlier in the lifecycle design process, in recent years, this approach has focused on speed and “good enough” software releases. Such a focus leads to potentially missing critical considerations unique to systems with embedded AI. Without adapting human systems engineering processes to account for the unique issues that AI introduce, especially in human-algorithm interactions, systems that leverage embedded AI are at a greater risk for significant problems, especially those that operate in safety-critical settings. Moreover, because of the unique roles humans play in safety-critical systems, either as designers, supervisors or teammates, it is especially important for Human Factors professionals, including user experience specialists, to be involved across the entire lifecycle.

The following sections will discuss how the requirements development and “ilities” processes will need to change, as well as current test, evaluation verification and validation approaches inherent in any systems engineering approach (National Academies of Sciences, 2022).

## 2. Requirements for AI development

Requirements development includes specifying high-level goals and functions for a desired system, including assigning responsibilities to various agents (humans or computer-based agents) (MITRE, 2014). A significant problem occurs in the requirements development process when engineers assume one system is more capable than it really is, which leads to a latent functionality gap.

AI can be powered by neural networks that work well in very narrow applications, but if such a system is presented with data that does not closely approximate the data with which it was originally trained, these algorithms can struggle to make sense of data with even slightly different presentations (Cummings, 2021). If requirements are developed that overestimate the capabilities of embedded AI and do not adequately consider the context and role of a human teammate (Tomsett et al., 2018), then system failure can occur.

The death of a pedestrian by the Uber self-driving car supervised by an inattentive safety driver (Laris, 2018) highlights such a disconnect. The designers of the system elected not to alert the safety driver when the underlying computer vision system struggled to correctly identify a potential threat. The system was also not designed to detect a distracted safety driver and these two design decisions, which were not identified as important requirements, directly led to the death of a pedestrian.

Identifying the points of AI brittleness is critical in the requirements stage because it means humans may need to adjust their cognitive work and unexpectedly take on new functions as a result of limitations in the underlying AI. Thus, an overreliance on AI capabilities will lead to a latent functionality gap, where humans may unexpectedly need to intervene for degraded AI but may not have the resources or time to do so. To this end, when considering how requirements development could or should change in the presence of AI, the following areas need dedicated focus^1^:

- Determine when, where and how AI-enabled technology is brittle.
- Determine what new functions and tasks are likely to be introduced as a result of incorporating brittle AI into complex systems.
- Determine those real-world constraints that could lead to AI brittleness.
- Reconcile the risk of AI brittleness and risk of human decision biases in determining appropriate functions, and whether exclusive roles or teaming between humans and autonomy best supports the overall mission, while providing safety buffers against inherent limitations.
- Investigate to what degree the risk of AI brittleness could affect human trust and how can appropriate trust be repaired, recalibrated, and maintained.
- Consider the influence of time pressure on human decision making for systems that leverage different kinds of AI.
- Determine the role of context in the various expected operational domains.
- Determine whether and how AI should be explainable/interpretable for actual users, especially those in time-critical settings and across different levels of training.
- Determine when the reasoning processes, decisions and limitations of embedded AI should be transparent, as well as the costs and benefits of making such information transparent.
- Delineate the circumstances and designs that humans can augment AI to mitigate inherent brittleness.
- Determine if acceptable levels of uncertainty should be characterized in the requirements process.
- Investigate whether systems should be designed so that humans and AI form complementary teams and determine any necessary redundancies for critical decisions.
- Determine if and how humans could aid in detecting adversarial attacks and whether such a function can cause workload to exceed acceptable limits.
- Map and communicate competency boundaries of both humans and AI-enabled systems so that degraded and potentially dangerous phases of operational systems can be avoided.

## 3. “ilities” of Embedded-AI systems

The next important step in any systems engineering effort is understanding the “ilities” relevant to a particular system. The “ilities” are non-functional developmental, operational and support requirements for a technology (MITRE, 2014). Common “ilities” include usability, reliability, suitability, maintainability, accessibility and sustainability.

To better understand how new ilities are emerging in the presence of AI, it is important to first understand the nature of bias in AI systems. There are multiple sources of bias that humans introduce into the design of AI systems, which include: (1) Bias from inappropriate data curation, (2) Bias in the design of algorithms, and (3) Bias in the interpretation of resulting algorithms (Cummings and Li, 2021).

In terms of data curation, it is well-established that bias can be inadvertently introduced into an AI system due to underlying data sample selection bias (e.g., Samimi et al., 2010; Gianfrancesco et al., 2018). However, there is substantially less research on how the actual curation of the data set affects outcomes. Even more problematic are errors made in actual data labeling, either by humans or machine-based labeling systems. One study looking at 10 major datasets cited over 100,000 times found a 3.4% average error rate across all datasets (Northcutt et al., 2021). These errors affect overall algorithm performance outcomes, and are pervasive in commercial language models and computer vision systems.

In addition to data curation, significant bias can be introduced into an AI system when the practitioner subjectively selects an AI algorithm and the associated parameters for an application. One recent study illustrated that there were at least 10 significant subjective decisions made by designers of machine-learning algorithms that could impact the overall quality of such models (Cummings and Li, 2021). There are currently no standards or accepted practices for how such points of bias and subjectivity could or should be evaluated or mitigated.

Lastly, the third major source of bias is the interpretation of complex statistics generated by AI, which is a well-documented point of weakness, even for experts (Tversky and Kahneman, 1974). Research efforts have recently attempted to make outputs more explainable (Chandler, 2020; Ferreira and Monteiro, 2020) or interpretable (Fernández-Loría et al., 2020). However, most of these efforts attempt to improve interpretability for experts and very little effort is aimed at helping users of AI-embedded systems.

The importance of this explainability gap cannot be understated, especially for users of time-pressured systems like those in transportation and military systems. Users likely have no idea that there are potentially many deeply-flawed assumptions and biases that could call into question any AI-generated results. This gap is also noteworthy because it impacts certification efforts, i.e., if external system evaluators who are not the AI creators cannot understand system outcomes, then they cannot develop appropriate confidence that such systems can meet the specified requirements.

Given these sources of bias, Table 1 outlines both how some traditional “ilities” will need to be adapted as well as new “ilities” that need to be considered. In addition to traditional usability concerns, there will need to be additional focus on making the limits of AI very transparent to users. The likely biggest change in the suitability category will be the need to address the notion of “concept drift,” also known as “model drift.” This occurs when the relationship between input and output data changes over time (Widmer and Kubat, 1996), making the predictions of such system irrelevant at best, or at worst, potentially dangerous. For example, if an embedded AI system analyzes images for real-time decisions and relies on an older training set of data to make a classification, this could lead to serious problems like incorrectly labeling a building as a weapons facility when it is actually a school. This drift represents a possible source of *dynamic* uncertainty that should be considered in determining whether and when a system meets its specified requirements.

**Table 1:** Adapting “ilities” for systems with embedded AI.

**“ility”**	**Needs**
**Traditional**	
Usability	• AI operational limitations and competency boundaries should be made transparent to users. • In appropriate settings, user should be able to conduct sensitivity analyses to explore a decision space, as well as the limitations. • Routine feedback about usability should be elicited from users, including post-software updates.
Suitability	• A process should be implemented that maps any operational dependencies created in the implementation of AI systems in order to determine what downstream processes could be negatively affected if an AI system is degraded or fails.
Sustainability	• A process for identifying changes in operations or environmental conditions that affect model outcomes should be implemented, including when retraining should occur for connectionist AI systems. • An incident repository should be created and routinely analyzed for all AI systems where users and supervisors can document erroneous, unusual and unexpected system behaviors. • A process for tracking software changes and possible unintended impact on either operations or human activity should be developed. • A process for tracking and documenting issues with concept drift as well as operator disuse, misuse or abuse of AI should be implemented.
**New**	
Auditability	• Data and resulting models should be periodically audited to uncover issues with suitability and sustainability, as well as possible issues with bias. • Automated tools will be needed to support humans conducting auditing tasks.
Passive vulnerabilities	• Adversarial machine learning vulnerabilities need to be identified and mitigated.

The notion of concept drift also affects the sustainability category, since the best way to prevent drift is to ensure the underlying data in any AI model adequately represents the current operation domain. To this end, there is a need to develop an AI maintenance community whose jobs would entail data base curation, data and model quality assurance, data applicability assessments, and working with testing personnel to determine downstream effects of problems. There have been recent efforts to crowd source such reports (e.g., Responsible AI Collaborative, 2022), but more formalized efforts are needed in the form of a dedicated AI maintenance workforce, which is an entirely new area of workforce development.

There is also a need to explicitly consider a new “ility” of *auditability*, which is the need to document and assess the data and models used in developing an AI-embedded system, in order to reveal possible biases and concept drift. While there have been recent research efforts in developing processes to better contextualize the appropriateness of datasets (Gebru et al., 2018), as well as model performance with a given data set (Mitchell et al., 2019), there are no known organized auditing efforts across companies or agencies. Safety-critical systems likely require a much higher level of auditability than pure software-based systems, and auditability would likely fall under the purview of the aforementioned AI maintenance workforce.

The last new “ility” category in Table 1 is called passive vulnerability. There is increasing evidence that AI systems trained on large data sets are especially vulnerable to forms of passive hacking, where the environment is modified in small ways to leverage vulnerabilities in the underlying machine learning algorithms. For example, adversarial machine learning was used to trick a Tesla into going 85 vs. 35 mph with a small amount of tape on a speed limit sign (O'Neill, 2020), and such scenarios also occur in natural language processing (Morris et al., 2020). Cybersecurity practices will need to revamped in the future to account for this new threat vector, which is unique to AI and the potential effect on safety is still an unknown.

## 4. Testing, evaluation, verification, and validation (TEVV) of AI systems

While requirements and -ilities generation provides designers of AI-embedded systems with the parameters of a system's architecture, equally important in the systems engineering process is the testing, evaluation, validation, and verification (TEVV) stage of technology development. TEVV ensures that the requirements are adequately met, along with highlighting where inevitable designs trades may have introduced unacceptable risk. Unfortunately, there is no industry or government consensus on what constitutes acceptable TEVV practices for embedded-AI systems. One recent report has highlighted military TEVV inadequacies for AI-embedded systems (Flournoy et al., 2020), and the National Transportation Safety Board has called for more rigorous oversight of testing and certification of autonomous vehicles (NTSB, 2019).

**Figure 1:**
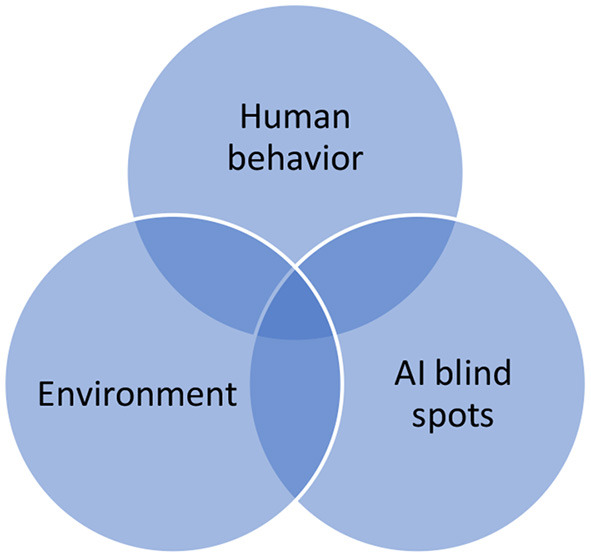
Sources of uncertainty in AI systems.

The primary reason that TEVV for AI systems is not well-understood is a current lack of methods to determine how such systems cope with known and unknown uncertainty. There are three primary sources of uncertainty in any AI system, as illustrated in Figure 1. Environmental uncertainty, like the impacts of weather, is a known source of uncertainty for both deterministic and non-deterministic systems. Similarly, human behavior for both actors in and external to a system can carry significant uncertainty. What is new in AI systems is the need to account for the variability, aka blind spots, in the embedded AI in such systems, and how these blind spots can lead to problems in human and AI performance in the actual operation of the systems.

While it is generally recognized that both commercial and government agencies need to adapt their testing practices to address the AI blind spot issues, there has been little tangible progress. Typical systems engineering approaches to testing generally include developmental tests at earlier stages of a technology's development, and then operational testing as a system matures. While this approach is reasonable for deterministic systems, it is simply not going to be sustainable for systems with embedded AI, as noted by others (Raz et al., 2021; Wojton et al., 2021).

One major issue is the constant updating of software code that is a necessary byproduct of agile software development. In such design approaches, software can change in seemingly small ways, but then lead to unexpected outcomes. Without principled testing, particularly for software than can have a derivative effect on human performance, the stage will be set for potential latent systems failures. Moreover, because software is typically continuously updated throughout the lifecycle of a system, it is not clear how testing should be adapted to catch the emergence of a problem in a system with embedded AI.

This staged approach to testing does not explicitly account for the need to test AI blind spots. Understanding that there are new sources of uncertainty that require rethinking of TEVV, especially as these sources of uncertainty relate to human work, new testbeds will be needed that allow for investigation of such uncertainties.

One of the core issues at the heart of AI experimentation is the role of simulation vs. real-world testing. Companies prefer to conduct the bulk of system experimentation in simulation for costs and scheduling reasons. Because uncertainty is a potential unknown unknown, which can come from the design of AI systems, the environment, and humans, much greater emphasis is needed in studying how much testing should be in simulation vs. the real world.

Given the changes that AI is and will continue to bring in both the design of systems as well as their use, there are several areas of inquiry that deserve more attention in terms of TEVV, which include:

- How should AI performance be measured, including individual behaviors as well as patterns of behaviors and emergent behaviors.
- How can known bias in the development of AI systems be measured and tested?
- How can unknown sources of bias be discovered?
- What methods could be developed to potentially reveal unknown sources of bias?
- The need for routine post-deployment testing, including person-in-the-loop evaluations, when meaningful software changes are made or environment conditions change. Additional work is needed to define “meaningful software changes.”
- How do human decision biases affect data curation, how can this be tested and what can be done to mitigate such biases?
- How could or should test cases be developed so that edge and corner cases are identified, particularly where humans are operating or could be affected by brittle AI?
- Testbeds should be developed that allow for multidisciplinary interactions and inquiry, and also that include enough real-world data to investigate the role of uncertainty as it relates to AI-blind spots.
- Given that changes occur on an almost continual basis in AI systems for both software and environmental conditions, identifying, measuring and mitigating concept drift is still very much an open question.
- How can humans certify probabilistic AI systems in real-world scenarios?
- Determining the appropriate degree of trust for systems that may not always behave in a repeatable fashion, like those with embedded AI, is critical for both people who operate and certify such systems.
- The development of a risk-based framework that balances algorithm-focused, embedded system and operational testing and what is the appropriate use of actual vs. simulation testing.
- A formulation for what constitutes unreasonable risks for different AI applications and how to test to such formulations, i.e., what may be considered an acceptable risk to a military self-driving tank is very different from that of a personally-owned self-driving car.

There are many groups in industry, government and academia working to better understand these issues, and there has been incremental progress (Freeman, 2020; DSTL, 2021; Tate, 2021). Progress tends to be narrow and function specific, for example, Waymo recently explained how it was testing its AI-enabled collision avoidance systems on cars (Kusano et al., 2022). While laudable, how to extend this approach more broadly to other systems or how to make this an industry-wide standard are still open questions. In addition, not only is more funding needed in the testing and certification space, research agencies need to understand that testing, safety and certification efforts are not simply peripheral activities that necessarily accompany research, but that they are legitimate research fields in and of themselves (Cummings et al., 2021).

## 5. Conclusion

The recent shift from predominantly hardware-based systems in complex settings with some embedded deterministic software to systems that heavily leverage probabilistic AI reasoning requires that typical systems engineering processes adapt, especially functional and non-functional requirements development and TEVV. While there has been a substantial body of literature that addresses how requirements should be developed for complex systems with embedded software, as well as how to test such systems, the bulk of this work assumes that the underlying software incorporates deterministic algorithms that perform the same way for every use.

However, AI that relies on probabilistic reasoning is brittle, and can fail in unexpected ways. The overestimation of AI capabilities could result in requirements that lead to systems that require human oversight in unexpected ways with little or no support. Such misapplication could also introduce new functionalities that result from an AI system reasoning in ways unanticipated by their designers. Moreover, there is little-to-no consensus on how such a system should be tested, how much simulation should play into acceptable test protocols and what constitutes “good enough” performance in safety-critical settings.

This work outlined 14 new considerations in the development of functional and task requirements for complex systems with embedded AI. These recommendations highlight the interconnectedness between uncertainty and AI, as well as the role one or more humans might need to play in the supervision and secure operation of such systems. In addition, 11 new or modified non-functional requirements, i.e., “ilities,” were also delineated that focus on the broad categories of usability, sustainability, and suitability while also introducing two new “ilities,” that of auditability and passive vulnerability. These new ilities highlight the need for a new workforce to specialize in AI maintenance.

In terms of TEVV, 10 areas of weakness were noted that need significantly more attention before developers can develop reasonable risk estimates of AI system performance. Companies and government agencies need to develop public-private partnerships to address AI testing and certification issues, especially those in safety critical settings. While government agencies have significant experience with formal system testing, commercial entities have the cutting-edge AI development experience, so combining their capabilities could advance the field far faster than if everyone attempts to develop their own testing and certification protocols.

Lastly, while AI may seem to predominantly belong in the domain of computer science, there are critical facets that need the expertise of people in the fields of human factors engineering, the social, cognitive, and physiological sciences, systems engineering, philosophy, public policy and law. If AI continues to be developed in a stove-piped manner, it could potentially have a dramatic negative impact in unexpected ways. While companies and government agencies can form interdisciplinary teams for specific projects, ultimately academia (and the associated funding agencies) needs to ensure that interdisciplinarity in research and education is both valued and rewarded so that students graduate with the interdisciplinary skill sets badly needed in government and industry.

## Data availability statement

The original contributions presented in the study are included in the article, further inquiries can be directed to the corresponding author.

## Author contributions

MC conceived and wrote the entire paper.

## Footnotes

^1^This is an extension of previous work (Ferreira and Monteiro, 2020).
